# Circular material flow of medication in the intensive care unit

**DOI:** 10.1186/s13054-025-05434-3

**Published:** 2025-05-20

**Authors:** Jasper Klasen, Silke Rijcks, Diederik Gommers, Jan Carel Diehl, Nicole Hunfeld

**Affiliations:** 1 Department of Adult Intensive Care, Erasmus MC University Medical Center, Dr. Molewaterplein 40, 3015 GD Rotterdam, The Netherlands; 2 Faculty of Industrial Design Engineering, Delft University of Technology, Delft, The Netherlands; 3 Department of Hospital Pharmacy, Erasmus MC University Medical Center, Rotterdam, The Netherlands

**Keywords:** Intensive care unit, Medication, Environmental sustainability, Material flow analysis, Healthcare waste, Circular economy

## Abstract

**Background:**

Intensive care units (ICUs) contribute significantly to healthcare's environmental footprint, with medications playing a major role. This study performed a comprehensive Material Flow Analysis (MFA) of medications in a large academic ICU to quantify material flows and identify opportunities for sustainability.

**Methods:**

A single-center MFA was conducted at a 50-bed ICU, analyzing all medications delivered in 2023. Medication and packaging components were weighed and categorized by active pharmaceutical ingredients (APIs), excipients, and packaging type. Total annual mass as well as daily medication and packaging waste per patient were calculated.

**Results:**

The annual medication inflow totaled 234,337 kg, including 194,411 kg of medication content (5287 kg APIs, 189,124 kg excipients) and 39,923 kg of packaging. APIs constituted only 2.3% of the total medication mass. On average, patients received 89.5 medication units daily, totaling 5.0 kg of medication and generating 1.7 kg of packaging waste. Waste outflow comprised 194,413 kg to the sewage system, 21,894 kg for incineration, and 18,030 kg recycled, consisting primarily of continuous renal replacement therapy (CRRT) bags.

**Conclusions:**

This MFA highlights significant opportunities to enhance ICU medication sustainability by targeting CRRT-related waste, optimizing fluid formulations to reduce excipient use, and minimizing packaging. These findings support the development of targeted interventions to reduce the environmental footprint of critical care.

**Supplementary Information:**

The online version contains supplementary material available at 10.1186/s13054-025-05434-3.

## Introduction

Intensive care units (ICUs) are identified as carbon hotspots due to their continuous operation, high resource utilization, and energy-intensive nature [1, 2]. Given this environmental burden, there is an urgent need to transform ICUs into more environmentally friendly healthcare systems [3–5]. Gaetani et al. showed that carbon emissions of ICUs range between 88 and 178 kg of CO_2_-equivalent per patient per day [5].

Hunfeld et al. performed a material flow analysis (MFA) that showed per-patient contributions of up to 17 kg of waste, 12 kg CO_2_-equivalent emissions and 300 L of water consumption [6]. This substantial impact highlights the necessity of targeted interventions in critical care settings.

Medication represents a significant share of healthcare's carbon footprint. In the Netherlands, pharmaceuticals and chemical products account for 41.2% of the healthcare sector's greenhouse gas (GHG) emissions [7]. At Erasmus MC University Medical Center, medication was responsible for 52.1 kilotons of CO_2_-equivalent in 2021, representing 41.6% of scope 3 emissions [8]. Scope 3 covers all indirect greenhouse gas emissions resulting from an organization's upstream and downstream activities (like producing purchased goods or treating waste) that fall outside its direct operational control (scope 1) or its purchased energy consumption (scope 2) [8]. Notably, while comprising only 5% of hospital beds, our ICU receives 18% of total hospital medication deliveries (based on analysis of internal pharmacy distribution records of 2023), highlighting the high medication intensity of the ICU.

Despite the widely recognized environmental burden of ICUs and medications, there remains a significant knowledge gap regarding the specific contribution of medications used in critical care. A deeper understanding of the current environmental footprint of the medication lifecycle, including the number of medications used, their packaging, and the composition of their active pharmaceutical ingredients (APIs), is essential. These data are critical to identify circular strategies. For this, the 10R-framework (see Supplementary Information 1) can be used; for instance, the recycling of continuous renal replacement therapy (CRRT) bags [9], or the reduction of packaging materials for medication.

The MFA of Hunfeld et al. evaluated the environmental impact of medical products used in the ICU, demonstrating the method’s utility for assessing resource use and waste generation in healthcare settings [6, 10]. However, that analysis did not specifically account for medications. To date, no studies have comprehensively assessed the environmental impact of ICU medications. Parvatker et al. investigated the cradle-to-gate GHG emissions for twenty anesthetic medications. However, this does not provide a complete overview of ICU medications and is not based on circular strategies [11]. Furthermore, the calculation of the total greenhouse gas emissions of all ICU medications requires complex life cycle assessments (LCAs) from cradle-to-grave, whilst the data needed to perform these LCAs are not publicly available [11].

The primary aim of this study is to quantify and characterize the material flows of medication in an academic ICU for adult patients for the year 2023. Secondary objectives include determining the ratios of active ingredients to excipients, as well as the weights of leaflets, primary packaging, and secondary packaging. These findings will help to develop circular strategies to reduce the ecological footprint of ICU medications.

## Methods

### Study design and scope

We conducted a single-center MFA (see Supplementary Information 2) of medications used in the ICU at Erasmus MC University Medical Center in 2023. The ICU is a 50-bed facility providing academic intensive care medicine in a mixed surgical and non-surgical setting, with capabilities for CRRT and extracorporeal membrane oxygenation (ECMO). It operates in line with national [12, 13] and international guidelines [14].

This study included all medications designated for patient use within the ICU that were part of the pharmacy's logistical chain and assigned an Anatomical Therapeutic Chemical (ATC) code [15]. Products with more than 250 deliveries in 2023 were weighed when available in this study, resulting in a potential coverage of 94.1% of total deliveries.

Tertiary packaging removed before medication entered the pharmacy, was excluded. Weights were assigned to single units of medication to align with delivery data and enable analysis at the individual medication level. CRRT-related medications were analyzed as a distinct subgroup due to their anticipated substantial contribution to the total material mass [6]. CRRT-related medication refers to infusion bags with fluids like citrate and dialysate commonly used in dialysis.

### Components

Medications consist of two primary components: The *APIs*, and *excipients*. APIs are the biologically active components of medications responsible for their therapeutic effects. Excipients are inactive substances (e.g., stabilizers, solvents, or fillers) included in drug formulations to ensure stability, bioavailability, or ease of administration. Together, APIs and excipients constitute the medication content [16]. Packaging was categorized into four types: *primary packaging*, which is the immediate container holding the medication, such as vials or blister packs; *secondary packaging*, which consists of external layers like boxes or cartons that provide additional protection; *package insert*, which are the leaflets included and *accessories*, which include supplemental materials (e.g., trays) (see Supplementary Information 3).

### Data collection

The following data were collected:

1. Delivery quantities for each medication product (based on article numbers) throughout 2023.
2. ICU admission days and patient numbers for 2023.
3. Medication details, including drug name, ATC code, dosage form (e.g., tablet, infusion fluid), and packaging type (e.g., blister, infusion bag).

### Mass of packaging components

Two researchers (JK and SR) weighed all available packaging components using three calibrated scales, each appropriate for different estimated weight ranges: a scale for products < 50 g with a precision of 0.01 g (Kitchenwell, The Netherlands); a scale for products 50–500 g with a precision of 0.1 g (Kitchenwell, The Netherlands) and a scale for heavier products with a precision of 1 g (Kitchenwell, The Netherlands).

The weight of primary packaging included the medication content (API + excipients). Net primary packaging weight was calculated by subtracting the API and excipient weights.

### Mass of API and excipients

API weights were obtained from summaries of product characteristics (SmPCs) provided by the Dutch Medicines Evaluation Board (MEB). Total medication weights were obtained from pharmaceutical companies. When total weights were unavailable, the following hierarchy was used:

1. Filling weight or volume provided by the manufacturer (assuming 1:1 weight-to-volume ratio for fluids).
2. Extractable fluid volume indicated on the label.
3. If this yielded no usable weight, the average medication weight of the corresponding dosage form was used (see Supplementary Information 4).

Excipient weight was calculated by subtracting the API weight from the total medication weight.

### Data analysis

The data analysis comprised the following steps:

1. Determination of API weight for each medication.
2. Calculation of excipient weight for each medication.
3. Determination of weights for primary and secondary packaging, package inserts, and accessories.
4. Calculation of mean weights for each component across weighed products. These mean weights were used to extrapolate to non-weighed products.
5. Calculation of total waste weight based on net empty packaging components.
6. Calculation of the weight and number of medications per patient per day (including CRRT).

All analyses were performed in R statistical software 4.4.1 (R Foundation for Statistical Computing, Vienna). Descriptive statistics included percentages, means, medians, interquartile ranges (IQRs), and ranges.

## Results

### Study population and pharmaceutical deliveries

The electronic health record (EHR) recorded 1,098,736 medication deliveries comprising 1800 unique products. We analyzed 395 products (22.0%) that were delivered more than 250 times in 2023, accounting for 1,033,498 deliveries (94.1% of total).

In 2023, 2569 patients were admitted to the ICU with a mean length of stay of 4.5 days. Of these, 289 patients (11.2%) underwent CRRT for a mean duration of 8.1 days.

### Distribution of pharmaceutical forms and classes

Primary packaging types showed a relatively uniform distribution, with blisters, ampoules, vials, infusion bags, and syringes each representing 16.2% to 22.9% of total deliveries (Table 1). The most frequently delivered medications were those affecting blood and blood-forming organs (27.0%, such as sodium chloride), followed by agents targeting the nervous system (20.7%, such as paracetamol) and cardiovascular system (18.3%, such as amiodaron).

**Table 1:** Baseline characteristics of medication delivered to the ICU in 2023

Variable	All medication deliveries		Medication with 250 or more deliveries	
	Different medications	Sum of deliveries	Different medications	Sum of deliveries
	*N* = 1800	*N* = 1 098 736	*n* = *395*	*n* = 1 033 498
Total, n. (%)				
Registered packaging type, n. (%)				
Blister	838 (46.6%)	251 715 (22.9%)	171 (43.3%)	211 769 (20.5%)
Ampoule	170 (9.4%)	200 665 (18.3%)	57 (14.4%)	193 927 (18.8%)
Vial	291 (16.2%)	218 454 (19.9%)	71 (18.0%)	211 322 (20.4%)
Infusion bag	69 (3.8%)	177 600 (16.2%)	34 (8.6%)	175 160 (16.9%)
Syringe	92 (5.1%)	201 669 (18.4%)	37 (9.4%)	200 228 (19.4%)
Sachet	45 (2.5%)	24 858 (2.3%)	3 (0.8%)	23 172 (2.2%)
Miscellaneous	295 (16.4%)	23 775 (2.2%)	22 (5.6%)	17 920 (1.7%)
ATC-code anatomical main group, n. (%)				
A—Alimentary tract and metabolism	258 (14.3%)	137 239 (12.5%)	66 (16.7%)	127 664 (12.4%)
B—Blood and blood forming organs	185 (10.3%)	296 672 (27.0%)	63 (15.9%)	289 932 (28.1%)
C—Cardiovascular system	286 (15.9%)	201 618 (18.3%)	90 (22.8%)	188 221 (18.2%)
D—Dermatologicals	67 (3.7%)	3 640 (0.3%)	4 (1.0%)	2 893 (0.3%)
G—Genito urinary system and sex hormones	48 (2.7%)	6 256 (0.6%)	8 (2.0%)	4 502 (0.4%)
H—Systemic hormonal preparations, excl. Sex hormones and insulins	77 (4.3%)	35 946 (3.3%)	19 (4.8%)	32 563 (3.2%)
J—Antiinfectives for systemic use	207 (11.5%)	101 044 (9.2%)	45 (11.4%)	92 493 (8.9%)
L—Antineoplastic and immunomodulating agents	91 (5.1%)	15 613 (1.4%)	11 (2.8%)	13 123 (1.3%)
M—Musculo-skeletal system	56 (3.1%)	15 435 (1.4%)	8 (2.0%)	13 581 (1.3%)
N—Nervous system	322 (17.9%)	227 325 (20.7%)	58 (14.7%)	215 794 (20.9%)
P—Antiparasitic products, insecticides and repellents	13 (0.7%)	615 (0.1%)	0 (0.0%)	0 (0.0%)
R—Respiratory system	83 (4.6%)	21 922 (2.0%)	10 (2.5%)	19 588 (1.9%)
S—Sensory organs	45 (2.5%)	2 242 (0.2%)	3 (0.8%)	1 693 (0.2%)
V—Various	40 (2.2%)	32 646 (3.0%)	10 (2.5%)	31 451 (3.0%)
Other^a^	22 (1.2%)	523 (0.0%)	0 (0.0%)	0 (0.0%)
Availability for measuring weight, n. (%)				
Product was available for weighing			317 (80.3%)	948 829 (91.8%)
Not available, alternative in dataset used as proxy			32 (8.1%)	45 326 (4.4%)
Not available, new medicine used as proxy			22 (5.6%)	16 795 (1.6%)
Not available, no proxy available			24 (6.1%)	22 548 (2.2%)

^a^Not all medication products that were delivered by the pharmacy have an ATC-code on file. These are mostly infusion fluids

Of the included 395 products, 78 (19.8%) were unavailable in the pharmacy during the measuring period (second quarter of 2024). For 54 of these products, a pharmaceutical equivalent proxy was used. For 24 of these products (6.1% of total included medicines, representing 2.2% of total included deliveries), no pharmaceutical equivalent was available as a proxy.

### Material flow analysis

**Fig. 1:**
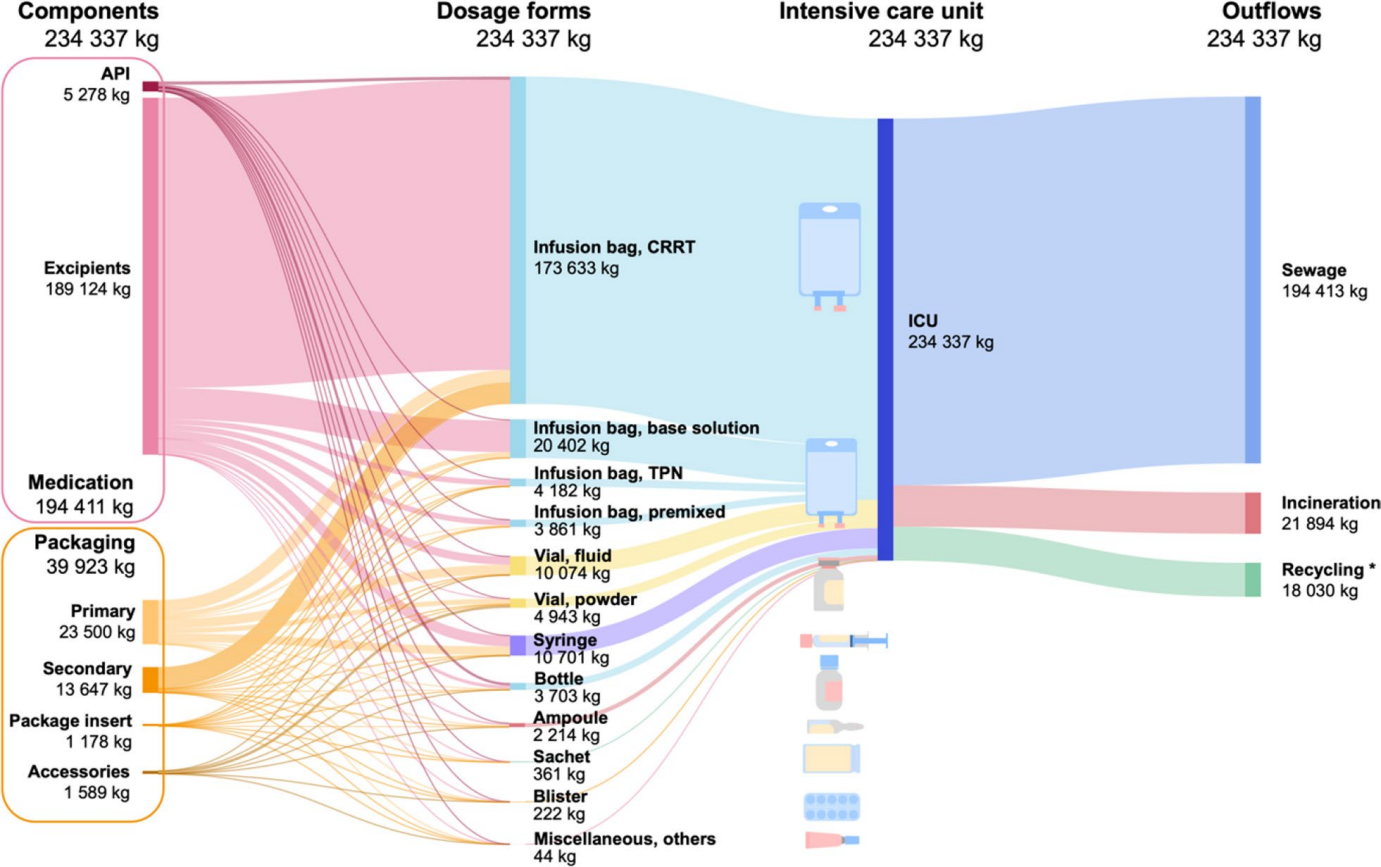
MFA of the ICU of Erasmus MC University Medical Center in 2023. Functional groups are shown on the left. The flow continues towards medication dosage forms and leaves the ICU with the outflow on the right side. ^*^In 2023, only packaging materials from CRRT related infusion bags were recycled, resulting in 45.2% of packaging mass being recycled. Other types of recycling (such as paper recycling) were not quantified and are therefore not included. Individual weights are rounded to the nearest kilogram

The total annual material flow consisted of 234,337 kg of medication. This is divided into 194,411 kg of medication content (5287 kg API, 189,124 kg excipients) and 39,923 kg of packaging materials (Fig. 1). The 39,923 kg of packaging consisted of primary packaging (23,500 kg), secondary packaging (13,647 kg), package inserts (1187 kg), and accessories (1589 kg). These weights correspond to the following dosage forms: CRRT related infusion bags 173,633 kg, base solution infusion bags 20,402 kg, Total Parenteral Nutrition (TPN) related infusion bags 4182 kg, premixed infusion bags 3861 kg, fluid-filled vials 10,074 kg, powder-filled vials 4943 kg, syringes 10,701 kg, bottles 3703 kg, ampoules 2214 kg, sachets 361 kg, blisters 222 kg and other forms 44 kg, totaling 234,337 kg. Of this, 194,413 kg exits in the sewage system, 21,894 kg gets incinerated, 18,030 kg is recycled (fully consisting of CRRT related infusion bags), resulting in 45.2% of packaging mass being recycled.

### API content analysis

Analysis of dosage forms revealed that fluid formulations consistently contained a lower mass percentage of APIs compared to solid formulations (see Supplementary Information 5). Among the most frequently delivered dosage forms, the API mass percentage varied considerably: tablets and capsules in blister packaging (n = 185) had a median of 10.0% (IQR 3.1% to 40.0%), fluids in ampoules (n = 60) 0.5% (IQR 0.1% to 6.1%), fluids in vials (n = 24) 1.0% (IQR 0.4% to 8.7%), powders in vials (n = 35) 83.3% (IQR 36.4% to 95.1%), and fluids in syringes (n = 32) 0.4% (IQR < 0.01% to 1.0%). Variability across dosage forms was most pronounced for fluid formulations, with ampoules and fluid-filled vials exhibiting broad interquartile ranges, while powders in vials and sachets showed less variability compared to others.

**Fig. 2:**
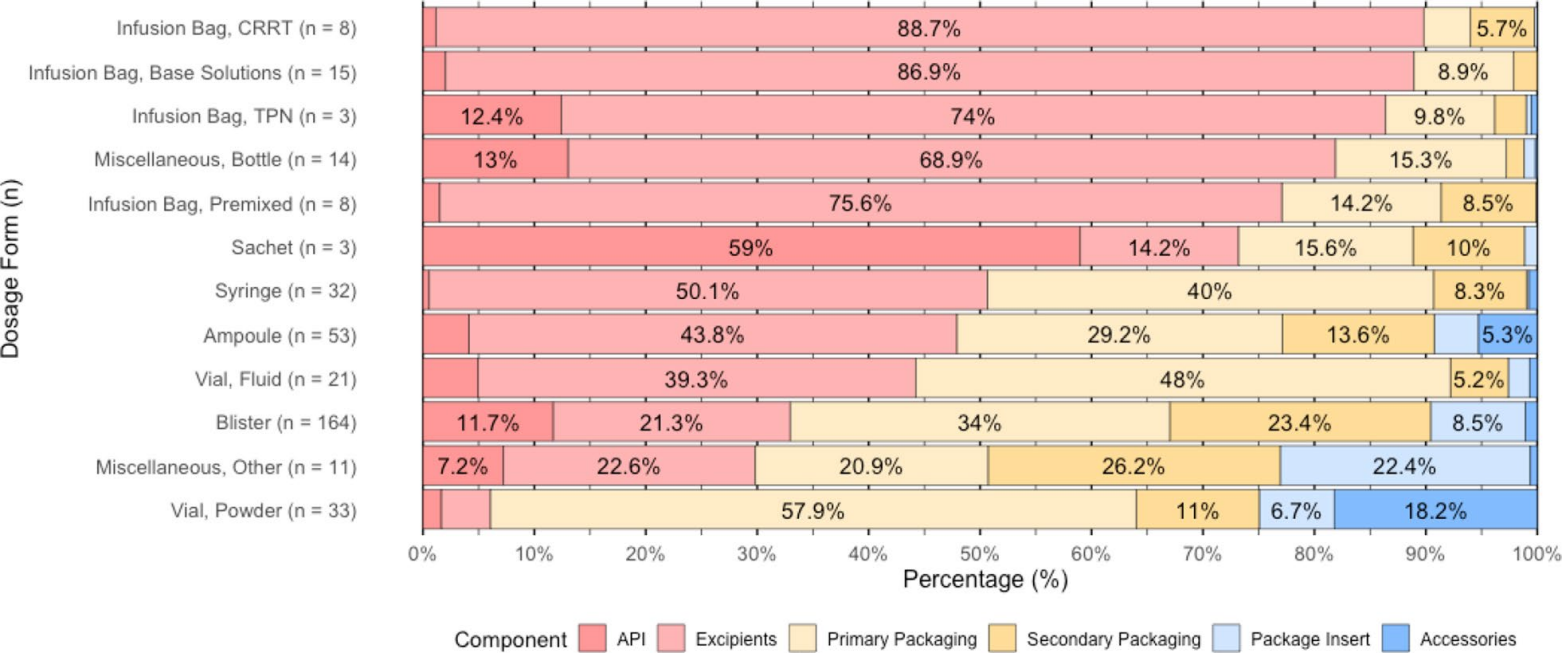
Proportional weight distribution of pharmaceutical components by dosage form. Ordered by share of medication to total weight (percentages smaller than 5% not labeled)

Figure 2 illustrates the distribution of individual weight components across dosage forms. Notably, accessories constitute 5.3% of ampoule weight, primarily consisting of trays to secure the ampoules during distribution, and 18.2% of powder-filled vial weight, mostly attributed to solvents in vials.

### Analysis of medications and waste per patient per day

**Fig. 3:**
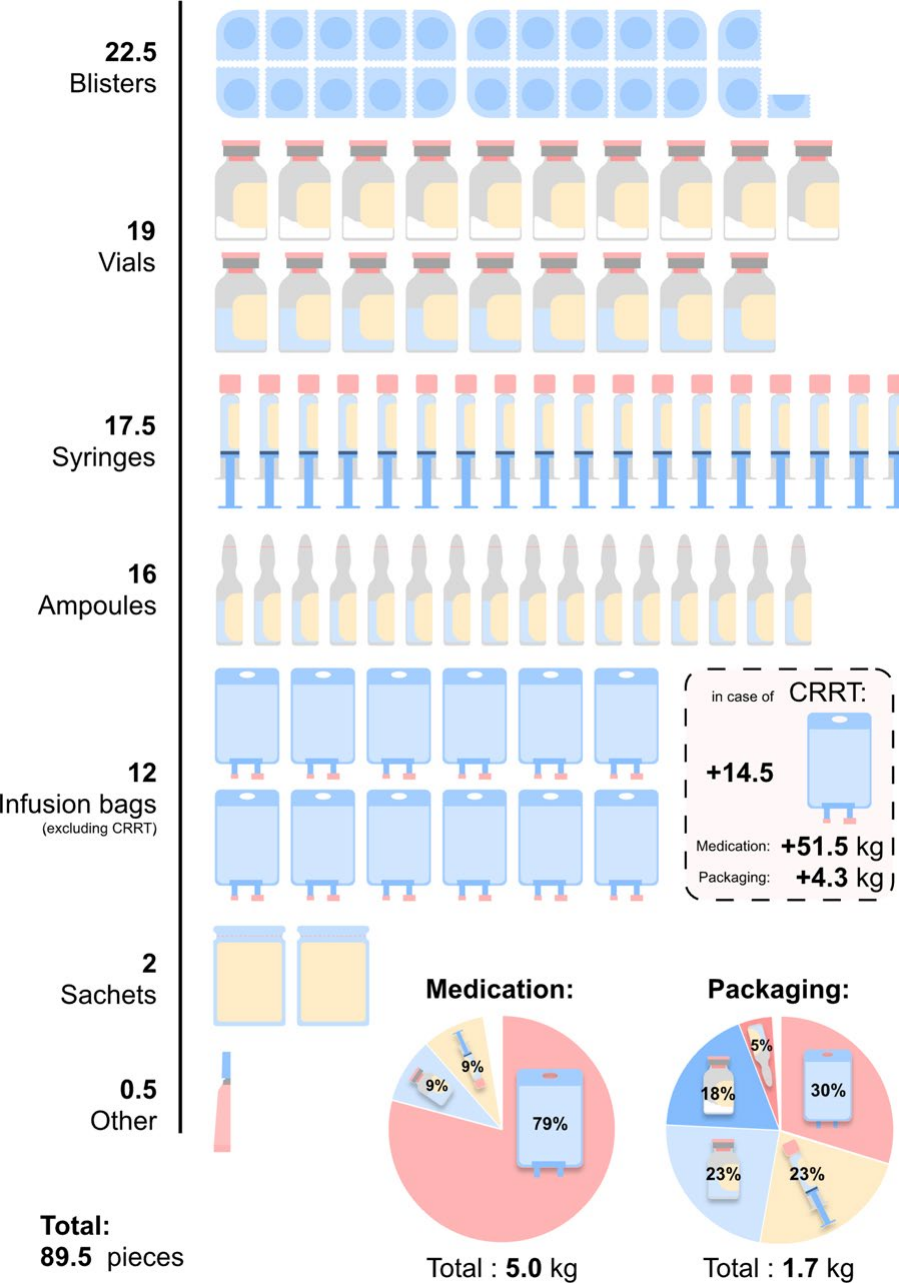
Overview of mass of medications and their associated packaging waste per ICU patient per day

On average, 89.5 medication items were delivered per patient daily (Fig. 3), including 22.5 blisters, 10 powder-filled vials, 9 fluid-filled vials, 17.5 syringes, 16 ampoules, 12 infusion bags (excluding CRRT), 2 sachets, and 0.5 other dosage forms. This corresponds to 5.0 kg of medication per day, with 79% of the weight attributed to infusion bags. The associated packaging adds up to 1.7 kg of waste, distributed as 30% from infusion bags, 23% from syringes, and 32% from vials. For patients receiving CRRT, an additional 14.5 infusion bags are used daily, contributing to 51.5 kg of fluids and 4.3 kg of packaging waste.

## Discussion

This study reveals that only a small fraction (2.3%) of the total medication-related mass comprises APIs, with the vast majority consisting of excipients and packaging materials. This disproportionate ratio underscores the importance of considering the API-excipient-packaging ratio in procurement procedures. This finding highlights that procurement decisions based solely on API may overlook significant material burdens associated with excipients and packaging. On average, ICU patients receive 89.5 medications daily, totaling 5.0 kg of medication and generating 1.7 kg of associated packaging waste. Of the 234,337 kg of medication delivered annually, 194,413 kg was discharged into the sewage system, 21,894 kg was incinerated, and 18,030 kg was recycled (primarily CRRT-related packaging). Quantifying the material flow in a 50-bed ICU provides a basis for reassessing existing medication protocols and developing targeted waste reduction strategies, ultimately enabling environmental sustainability in critical care.

This study highlights several key areas for intervention. First, the substantial contribution of CRRT to the overall material flow (74.1% of total inflow) necessitates targeted strategies to reduce waste in this area. This could include exploring alternative CRRT modalities that minimize fluid and material use or optimizing current protocols to reduce waste. Second, the low API mass percentage in fluid formulations, particularly in syringes (1.3%) and ampoules (5.8%), suggests potential for developing more concentrated formulations. This could significantly reduce waste, especially given the increasing reliability and widespread use of infusion pumps in ICUs. Third, the significant mass associated with fluid formulations also highlights the potential impact of clinical practice choices, such as favoring oral administration when appropriate, as promoted by initiatives like the “paracetamol challenge” (encouraging oral administration when clinically appropriate) [17]. Fourth, the annual disposal of 1187 kg of package inserts presents an opportunity for improvement, as this information is readily available online. The 2024 pilot program for electronic product information (ePI) in the Netherlands, part of a broader EU initiative, is a promising development that will eliminate the need for many paper package inserts, including those for the medications analyzed in this study [18, 19].

This MFA quantified the outflow pathways, indicating that 194,413 kg of the medication mass (primarily water and excipients from fluid formulations) is released into the sewage system annually. While a detailed analysis of the environmental fate of these substances is beyond the scope of this study, the presence of pharmaceuticals in wastewater is a recognized environmental concern, potentially contributing to water source contamination, aquatic ecosystem disruption, and antimicrobial resistance [20]. However, it is important to contextualize this outflow figure within Dutch wastewater management practices. Regional Water Authorities employ advanced treatment processes for municipal and hospital effluent, which remove the vast majority of APIs (filtering out 92.3%), although complete elimination remains a challenge [21]. Another Dutch academic hospital contributed only 3.5% to the pharmaceutical wastewater load in the region, with two-thirds of that removed by treatment [22]. Understanding the precise environmental impact of the medication outflow from our ICU is a topic for future research.

Besides the sewage outflow, the MFA clearly quantified waste streams amenable to immediate circularity interventions. Our ICU's practice of recycling CRRT-related packaging materials in 2023 demonstrates that a targeted intervention can significantly reduce the amount of incinerated waste, in this case cutting the total packaging mass sent for incineration by 45.2% [9]. The analysis further identified other substantial packaging components currently incinerated, such as the 7697.5 kg of annual waste from glass vial packaging (35.2% of total incinerated packaging waste), representing another major material stream where future recycling efforts could be of substantial impact.

Despite its comprehensive scope, this study has several limitations. First, although MFA is a valid proxy for environmental harm when high-quality LCA data are unavailable [10], it is less precise. LCAs, especially when conducted in adherence to ISO norms 14,040 and 14,044, provide the most precise method for assessing environmental impact. However, cradle-to-gate data for all 1,800 medications used in the ICU are unavailable, and conducting separate LCAs for each medication would require immense time and resources. An alternative approach, such as spend-based LCA (based on financial pricing), was also considered but deemed unreliable due to the weak correlation between pharmaceutical pricing and production costs.

Second, as a single-center study conducted in the Erasmus MC University Medical Center ICU, the generalizability of our findings may be limited. However, as one of the largest ICUs in the Netherlands, it is likely representative of other academic ICUs in similar healthcare settings.

Third, the exclusion of tertiary packaging (primarily cardboard, which is routinely recycled) may slightly underestimate total upstream waste, but its impact on the overall findings is likely minimal.

Fourth, medication delivery data to the ICU, rather than administration data, were used in this analysis. This methodological choice was necessitated by the lack of patient-specific administration records for CRRT and infusion bags, making delivery data the most reliable source of information. Consequently, the transfer of medications between ICU and general wards upon patient admission and discharge were not considered and fall outside the scope of this study.

Finally, this MFA focused specifically on material flows and did not quantify the energy or water consumption. It is important to acknowledge that the relative environmental impact of energy consumption versus medication use is highly context-specific, influenced by factors such as building infrastructure, energy efficiency initiatives, and the carbon intensity of the local energy grid.

This study's strengths include its comprehensive analysis of medication material inflows and outflows. By employing a detailed MFA that included weighing 94.1% of total medication deliveries, we provide a nuanced examination of medication use in the ICU setting. Most notably, this study is the first to quantify the proportion of APIs (2.3%) relative to excipients and packaging in ICU medications, providing a crucial insight for future sustainability initiatives.

### Recommendations

The study highlights the value of MFA in assessing the environmental impact of medications. The linear lifecycle of most ICU medications, characterized by resource-intensive production, single-use packaging, and disposal of packaging through incineration, hinders circularity. To promote circularity, sustainable interventions are needed. These interventions can be linked to the different R-strategies (see Supplementary Information 1) and categorized as follows:

1. *Medication-related strategies*:
2. Prioritize oral formulations (tablets and capsules) over intravenous formulations whenever clinically appropriate (Rethink).
3. Develop and promote the use of more concentrated fluid formulations to reduce transport, storage, and packaging waste (Refuse, Rethink, Reduce).
4. Explore and implement alternatives to traditional CRRT that minimize fluid and material use (Rethink, Reduce).
5. *Packaging-related strategies*:
6. Optimize packaging designs to minimize material use and waste (Reduce).
7. Reduce or eliminate the use of package inserts in ICU settings, leveraging electronic resources (Reduce, Refuse).
8. Expand recycling programs to include additional materials such as paper, glass vials, infusion bags, and other suitable packaging components, applying circular economy principles to ICU waste management (Recycle).

## Conclusion

This study quantifies the annual medication material flow in a 50-bed academic hospital ICU. Of 234,337 kg of medication delivered in one year to the ICU, 194,413 kg ends up in the sewage system, 21,894 kg gets incinerated, and 18,030 kg is recycled. CRRT accounts for 74.1% of the total inflow, highlighting its disproportionate contribution to material flow. APIs constitute only 2.3% of total medication mass, with fluid formulations, particularly syringes (1.3% API) and ampoules (5.8% API), exhibiting the lowest API content, underscoring the dominance of excipients. Packaging materials, including 1187 kg of annual package inserts, represent a significant waste burden, with only CRRT-related packaging (18,030 kg) being recycled. These results establish a baseline for sustainability interventions in critical care. Thus, our data highlight key areas for sustainable interventions, including CRRT bags, fluid formulations, and the reduction of packaging waste. Future efforts should prioritize innovations in packaging design, implementing reduce and reuse strategies for systems, and reducing the ecological footprint of critical care.

## Supplementary Information

Supplementary Material 1.

Supplementary Material 2.

Supplementary Material 3.

Supplementary Material 4.

Supplementary Material 5.

## Data Availability

The data that support the findings of this study are not publicly available due to confidentiality agreements with pharmaceutical companies and the inclusion of sensitive operational information from the hospital, but are available from the corresponding author on reasonable request. Data are located in controlled access data storage at the Erasmus MC.
