# Supplementary material for: Circular material flow of medication in the intensive care unit
Source: Crit Care. 2025 May 20;29:205. doi: 10.1186/s13054-025-05434-3 (PMC12093750; doi:10.1186/s13054-025-05434-3)
Supplement: Supplementary file 1 — Supplementary Material 1. [file 13054_2025_5434_MOESM1_ESM.docx]

**Supplementary Information 1** The 10R Circular Economy strategies depicted along the value hill [1]

#
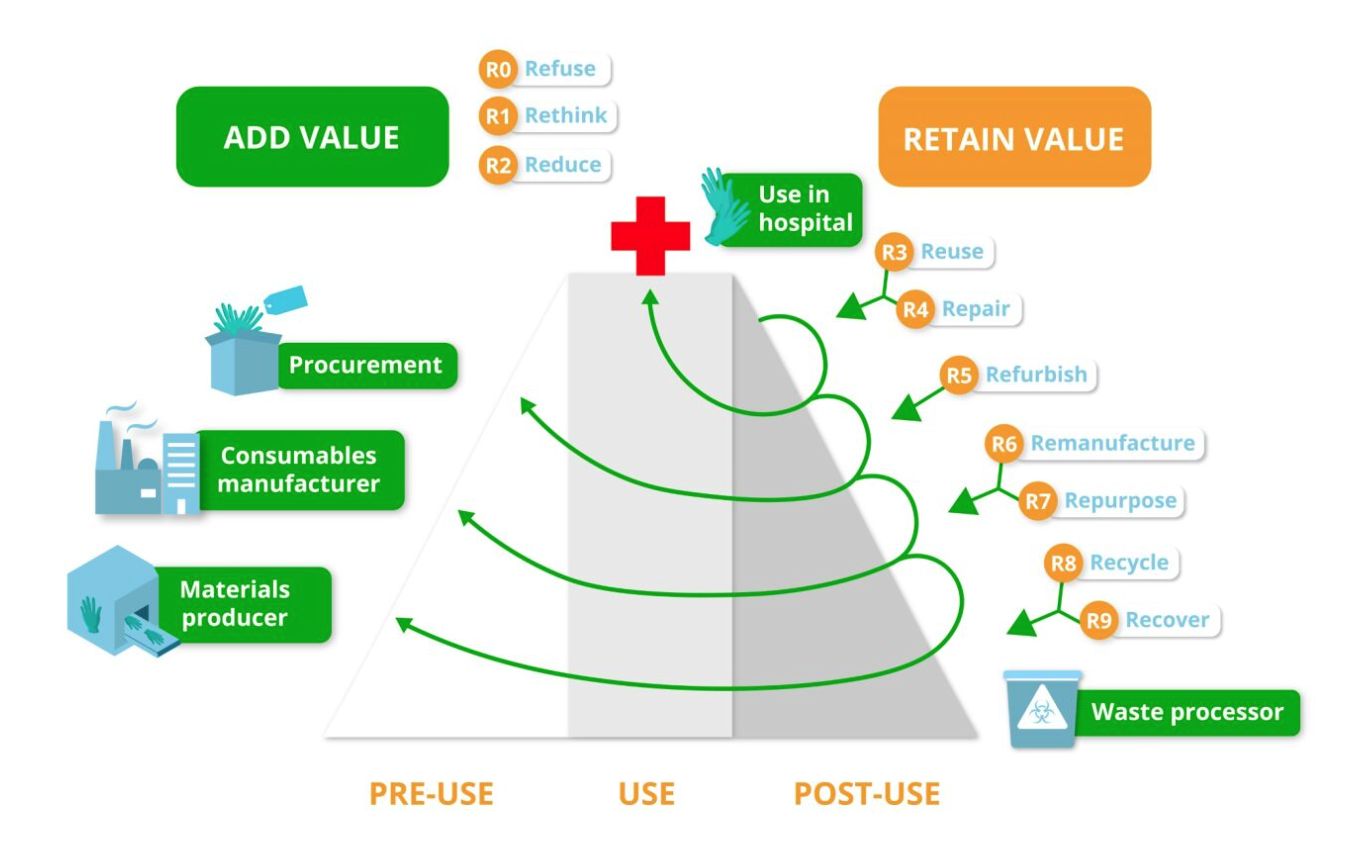


The 10Rs provide a framework for sustainable practices in healthcare. **Refuse** involves avoiding unnecessary medications or single-use packaging where possible. **Rethink** encourages alternative approaches, such as using other dosage forms. **Reduce** focuses on minimizing the amount of medication and packaging used. **Reuse** is about reusing products, such as by reusing pill bottles of tertiary packaging. **Repair** applies to maintaining and fixing reusable devices like syringe pumps to extend their lifespan. **Refurbish** involves restoring medical devices, such as infusion systems, to operational standards. **Remanufacture** can be seen in rebuilding medical equipment with certified components to match original specifications. **Repurpose** might mean finding new uses for certain materials, like turning expired or unused medications into training tools. **Recycle** ensures proper processing of medication packaging, such as glass vials or CRRT infusion bags, and **Recover** emphasizes extracting energy or materials from medical waste, by incineration instead of using landfills.

# Literature

1. Hunfeld N, Diehl J, de Zeeuw S, Gommers D, Raaij E. The Green Intensive Care: From Environmental Hotspot to Action. ICU Management & Practice. 2023;23(3).
