# Supplementary material for: Circular material flow of medication in the intensive care unit
Source: Crit Care. 2025 May 20;29:205. doi: 10.1186/s13054-025-05434-3 (PMC12093750; doi:10.1186/s13054-025-05434-3)
Supplement: Supplementary file 2 — Supplementary Material 2. [file 13054_2025_5434_MOESM2_ESM.docx]

**Supplementary Information 2** Material Flow Analysis [2, 3]

Definition and Core Principles:

Material Flow Analysis (MFA) is a systematic methodology used to track and quantify the flows of materials and resources entering, circulating within, and exiting a precisely defined system over a specific time period. The core objective is to generate a comprehensive overview of material throughput, typically expressed in units of mass (e.g., kilograms or tons). MFA operates on the fundamental principle of mass balance (the total mass of materials entering a system must equal the total mass leaving the system).

System Boundaries:

A critical first step in MFA is establishing clear system boundaries. These boundaries define the scope of the analysis and include:

- Spatial Boundaries: The physical limits of the system under investigation (e.g., a specific Intensive Care Unit (ICU), an entire hospital building, or a network of facilities).
- Temporal Boundaries: The time period over which the flows are analyzed (e.g., a single day, a week, or commonly a full year for systems like hospitals).
- Process Boundaries: The specific activities included within the analysis (e.g., procurement, storage, patient care activities, waste management).
- Material Scope: The specific types of materials being tracked (e.g., medical supplies, pharmaceuticals, water, energy, various waste categories).

Other key definitions:
In MFA, flows quantify the movement of materials between different processes or across the system boundary, measured as mass per unit time (e.g., kilograms per day). These flows are distinguished into inputs, which are materials entering the defined system from external sources (like deliveries of medical supplies, energy, or water), and outputs, which are materials leaving the system boundary to the environment or other systems (such as wastewater, emissions, or various types of waste). Analyzing input flows helps evaluate resource dependency and opportunities for reduction, while examining output flows is essential for assessing environmental impacts and optimizing waste management strategies.

Methodology in Healthcare:

Conducting an MFA in a healthcare setting involves:

1. Defining System Boundaries: Clearly outlining the spatial, temporal, process, and material scope relevant to the research question (e.g., focusing on consumable use and waste generation within an ICU over one year).
2. Identifying Flows and Stocks: Mapping the movement of key materials – inputs (supplies, energy, water), outputs (waste streams, wastewater), internal flows (between departments), and stocks (inventory).
3. Quantifying Flows and Stocks: Collecting data to measure the mass of each identified flow and stock. Data sources often include procurement records, inventory systems, waste logs, utility bills, and sometimes direct measurements or waste audits. Mass balance calculations are applied to validate data consistency. Data quality and availability can be challenging in complex healthcare environments, requiring careful documentation of assumptions and uncertainties.
4. Analyzing and Interpreting Results: Analyzing the quantified data to understand the system's material metabolism. This often involves visualization (e.g., Sankey diagrams) and calculating key indicators (e.g., material intensity per patient day, waste generation rates).

Applications and Insights in Healthcare:

MFA provides valuable insights for improving sustainability in healthcare:

- Identifying Hotspots: It is well-suited to pinpoint areas of high material consumption, resource inefficiency, or significant waste generation within the system (e.g., identifying specific procedures or departments with high disposable item usage).
- Waste Management: Provides a detailed profile of waste streams (e.g., general, infectious, pharmaceutical, plastic waste), enabling targeted reduction strategies and improvements in segregation and recycling.
- Environmental Performance: Establishes a quantitative baseline for tracking progress towards sustainability goals and understanding the material implications of different practices (though assessing broader environmental impacts like carbon footprint often requires integration with methods like Life Cycle Assessment - LCA).
- Circularity: Supports the transition towards a circular economy by identifying opportunities for *reuse, repair, refurbishment,* or *recycling* of materials and products within the healthcare system.

Strengths and Limitations:

The primary strength of MFA lies in its quantitative, systematic approach to understanding resource use and waste, making it ideal for identifying resource depletion hotspots, improving system circularity, and monitoring progress towards reduction targets. However, MFA focuses primarily on mass flows. Evaluating the specific environmental impacts associated with these flows (e.g., toxicity, climate change potential) requires additional analysis, often through LCA. Furthermore, comparing absolute MFA results between different studies can be challenging due to potential variations in system boundaries and methodologies, highlighting the need for clear boundaries.

Context in Healthcare:

While MFA is a relatively newer methodology in the healthcare sector compared to industrial ecology, its application is growing. As an example, our previous MFA conducted in an ICU setting revealed a significant material throughput, quantifying an average daily use of approximately 17 kg of medical products per patient, of which 11.9 kg were liquids [3]. This highlights the substantial resource flows even within specialized hospital units and underscores the potential for MFA to guide sustainability interventions.

# Literature

2. Brunner PH, Rechberger H. Practical Handbook of Material Flow Analysis. 2004.

3. Hunfeld N, Diehl JC, Timmermann M, van Exter P, Bouwens J, Browne-Wilkinson S, et al. Circular material flow in the intensive care unit-environmental effects and identification of hotspots. Intensive Care Med. 2023;49(1):65-74. doi: 10.1007/s00134-022-06940-6.
