# Supplementary material for: Circular material flow of medication in the intensive care unit
Source: Crit Care. 2025 May 20;29:205. doi: 10.1186/s13054-025-05434-3 (PMC12093750; doi:10.1186/s13054-025-05434-3)
Supplement: Supplementary file 3 — Supplementary Material 3. [file 13054_2025_5434_MOESM3_ESM.docx]

**Supplementary Information 3** System Boundaries, Pharmaceutical Components and Packaging Components for Pharmaceuticals


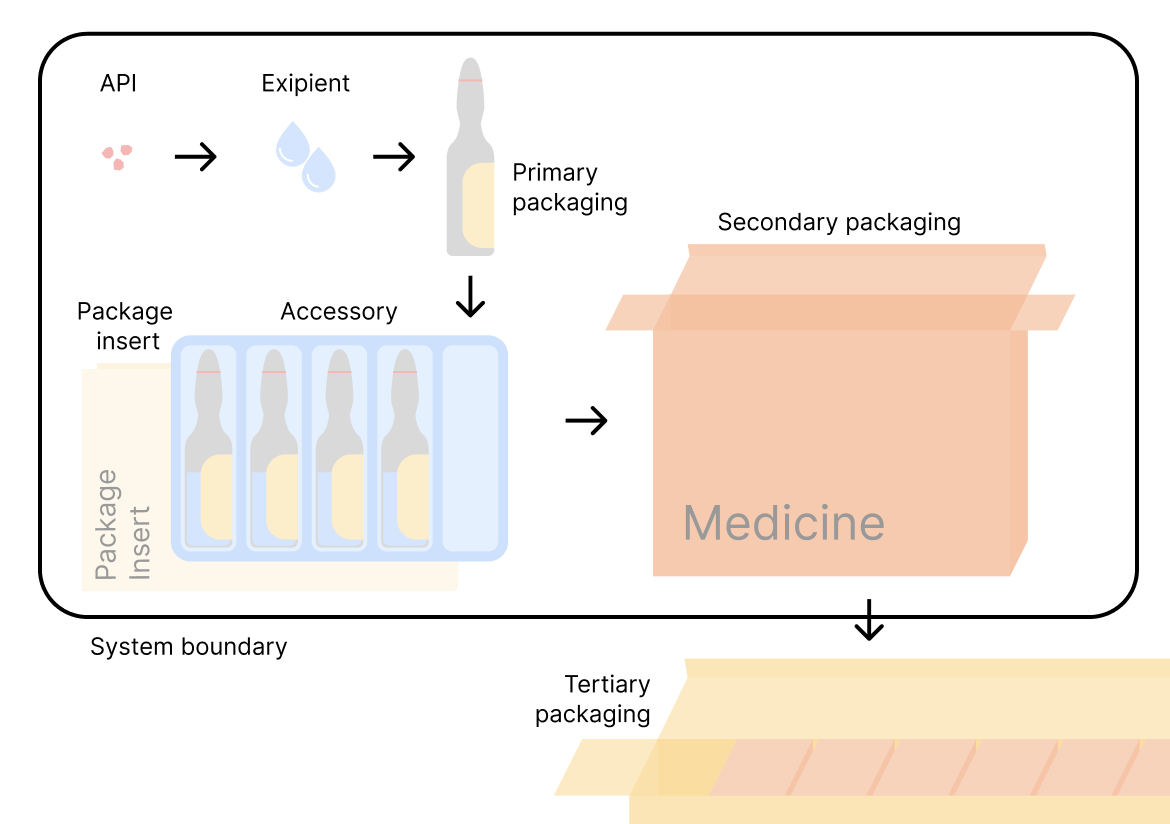


**Primary Packaging**: This is the packaging material that comes into direct contact with the medicine formulation (the mixture of API and excipient). Examples include vials, ampoules, blister packs, or bottles.

**Accessory**: An additional item included in the packaging needed for the administration or use of the medicine, such as a syringe, dropper, measuring spoon, or applicator.

**Package Insert**: A printed leaflet included in the package that contains important information about the medicine, including instructions for use, dosage, warnings, side effects, and storage conditions.

**Secondary Packaging**: This is the outer packaging that groups the primary packaging along with any accessories and the package insert. It provides additional protection and carries branding and regulatory information. A cardboard box is a common example.

**System Boundary:** This line defines the scope of the process being considered. Everything inside the boundary is part of this specific study.

**Tertiary Packaging:** This is used for bulk handling, transport, and shipping. It groups multiple secondary packages (the individual "Medicine" units) together, such as in a large shipping carton or on a pallet.
