# Supplementary material for: Circular material flow of medication in the intensive care unit
Source: Crit Care. 2025 May 20;29:205. doi: 10.1186/s13054-025-05434-3 (PMC12093750; doi:10.1186/s13054-025-05434-3)
Supplement: Supplementary file 4 — Supplementary Material 4. [file 13054_2025_5434_MOESM4_ESM.docx]

| **Main Category** | **Total Medicine (g)** | **API (g)** | **Excipients (g)** | **Total Packaging (g)** | **Primary Packaging (g)** | **Secondary Packaging (g)** | **Package Insert (g)** | **Accessories (g)** |
| --- | --- | --- | --- | --- | --- | --- | --- | --- |
| ampoule (n = 53) | 5.91 | 0.51 (0.07 -0.95) | 5.40 (3.93 -6.87) | 6.42 | 3.60 (3.07 -4.13) | 1.68 (1.36 -2.00) | 0.49 (0.38 -0.60) | 0.65 (0.47 -0.83) |
| blister (n = 164) | 0.31 | 0.11 (0.08 -0.14) | 0.20 (0.16 -0.23) | 0.63 | 0.32 (0.28 -0.36) | 0.22 (0.21 -0.24) | 0.08 (0.07 -0.09) | 0.01 (-0.00 -0.01) |
| infusion bag, CRRT (n = 8) | 3533.75 | 45.91 (8.58 -83.23) | 3487.84 (1819.04 -5156.65) | 400.56 | 164.50 (110.65 -218.35) | 225.96 (103.50 -348.42) | 10.10 (3.02 -17.18) |  |
| infusion bag, TPV (n = 3) | 1666.67 | 239.61 (-415.50 -894.72) | 1427.06 (-61.92 -2916.04) | 262.62 | 188.67 (47.13 -330.20) | 54.71 (-46.14 -155.55) | 8.98 | 10.26 (-13.33 -33.85) |
| infusion bag, base solutions (n = 15) | 373.54 | 8.51 (4.36 -12.67) | 365.03 (165.15 -564.92) | 46.49 | 37.59 (25.89 -49.28) | 8.86 (4.19 -13.52) | 0.04 (-0.03 -0.11) |  |
| infusion bag, premixed (n = 8) | 85.62 | 1.67 (0.18 -3.16) | 83.95 (43.22 -124.69) | 25.42 | 15.82 (12.79 -18.84) | 9.48 (1.03 -17.92) | 0.12 (-0.07 -0.30) |  |
| miscellaneous, bottle (n = 14) | 181.04 | 28.84 (1.63 -56.05) | 152.20 (53.41 -251.00) | 40.01 | 33.86 (18.91 -48.81) | 3.53 (0.22 -6.85) | 2.26 (-0.83 -5.36) | 0.36 (-0.42 -1.13) |
| miscellaneous, other (n = 11) | 1.90 | 0.46 (-0.23 -1.15) | 1.44 (0.06 -2.82) | 4.47 | 1.33 (0.60 -2.07) | 1.67 (0.22 -3.12) | 1.43 (-0.48 -3.35) | 0.04 (-0.04 -0.13) |
| sachet (n = 3) | 5.71 | 4.60 (-14.97 -24.17) | 1.11 (-3.01 -5.24) | 2.09 | 1.22 (0.23 -2.21) | 0.78 (-0.53 -2.09) | 0.09 (-0.04 -0.22) |  |
| syringe (n = 32) | 25.79 | 0.28 (0.08 -0.47) | 25.51 (17.41 -33.61) | 25.11 | 20.37 (15.58 -25.16) | 4.25 (2.51 -5.99) | 0.12 (0.05 -0.20) | 0.37 (-0.17 -0.90) |
| vial, fluid (n = 21) | 39.56 | 4.42 (-1.26 -10.10) | 35.14 (20.88 -49.40) | 49.88 | 42.91 (26.12 -59.70) | 4.64 (3.14 -6.14) | 1.72 (0.86 -2.57) | 0.61 (-0.04 -1.26) |
| vial, powder (n = 33) | 2.52 | 0.69 (0.31 -1.07) | 1.83 (-1.28 -4.94) | 39.00 | 24.06 (17.18 -30.94) | 4.58 (2.63 -6.53) | 2.80 (1.58 -4.02) | 7.56 (-1.52 -16.65) |

**Supplementary Information 4** Average weight components of different dosage forms per individual delivery unit
