# Supplementary material for: Circular material flow of medication in the intensive care unit
Source: Crit Care. 2025 May 20;29:205. doi: 10.1186/s13054-025-05434-3 (PMC12093750; doi:10.1186/s13054-025-05434-3)
Supplement: Supplementary file 5 — Supplementary Material 5. [file 13054_2025_5434_MOESM5_ESM.docx]

# **Supplementary Information 5** API ratio of different dosage forms

| **Category Type** | **Dosage Form (n = 378)** | **Median API**  **%** | **Min API %** | **Max API %** | **Q1 API %** | **Q3 API %** | **IQR API %** | **CV API %** |
| --- | --- | --- | --- | --- | --- | --- | --- | --- |
| Fluid dosage forms | syringe (n = 32) | 0.4 | <0.01 | 10.0 | <0.01 | 1.0 | 1.0 | 192.6 |
|  | infusion bag (n = 36) | 1.0 | <0.01 | 26.1 | 0.9 | 5.0 | 4.1 | 158.2 |
|  | ampoule (n = 60) | 0.5 | <0.01 | 100.0 | 0.1 | 6.1 | 6.0 | 255.1 |
|  | vial, fluid (n = 24) | 1.0 | <0.01 | 78.7 | 0.4 | 8.7 | 8.2 | 202.7 |
| Solid dosage forms | blister (n = 185) | 10.0 | <0.01 | 94.7 | 3.1 | 40.0 | 36.9 | 112.1 |
|  | sachet (n = 6) | 54.4 | 0.3 | 100.0 | 18.8 | 93.3 | 74.5 | 81.3 |
|  | vial, powder (n = 35) | 83.3 | 0.3 | 100.0 | 36.4 | 95.1 | 58.7 | 53.0 |
